# Maternal healthcare insurance ownership and service utilisation in Ghana: Analysis of Ghana Demographic and Health Survey

**DOI:** 10.1371/journal.pone.0214841

**Published:** 2019-04-25

**Authors:** Sanni Yaya, Feng Da, Ruoxi Wang, Shangfeng Tang, Bishwajit Ghose

**Affiliations:** 1 School of International Development and Global Studies, University of Ottawa, Ottawa, Canada; 2 School of Pharmacy, Tongji Medical College, Wuhan, China; 3 School of Medicine and Health Management, Tongji Medical College, Wuhan, China; 4 Institute of Nutrition and Food Science, University of Dhaka, Dhaka, Bangladesh; University Hospital of Jena, GERMANY

## Abstract

**Objectives:**

Previous studies have attempted to assess the role of health insurance on health care utilization in African settings. However, there is limited evidence on the effects of health insurance on use of maternal health care. In the present study our objective was to measure the prevalence of insurance ownership, types of services covered by the insurance and the association of insurance ownership with the utilization of respective maternal health services in Ghana.

**Methods:**

This study was based on nationally representative Demographic and Health Survey in Ghana (GDHS 2014) encompassing 4,293 mothers aged 15–49 years. Outcome variables were use of early antenatal care (ANC), facility delivery, and postnatal care (PNC) for mothers and children, and the explanatory variables were insurance coverage for these services. Associations were analysed using logistic regression models whilst controlling for potentially confounding variables.

**Results:**

Prevalence of health insurance ownership was 66.8% (95%CI = 64.5–68.9) with significant socioeconomic disparities. The prevalence was higher particularly among women who were urban residents, had higher educational and wealth status. In general, insurance coverage for services such as ANC, childbirth and postnatal care was higher in rural areas, but that of cash benefit was higher in urban areas. Findings of multivariate analysis indicated that women who had their ANC services covered had significantly higher odds of attending at least one and four ANC visits, as well as receiving PNC for child. Insurance coverage for childbirth services showed significant association with the PNC for child, not with choice of health facility delivery. Women who had cash benefit were twice as likely to use early ANC visit (OR = 2.046, p<0.05), facility delivery (OR = 1.449, p<0.05), and PNC for mother (OR = 1.290, p<0.05).

**Conclusion:**

Overall prevalence of health insurance coverage has increased since 2008, with significant disparities across demographic and socioeconomic groups. Insurance ownership for different types of maternal health services showed positive association with service uptake, with exceptions for place of delivery, indicating that insurance coverage alone may not be able to promote facility delivery. More studies are required to measure the progress in maternal healthcare utilisation through the insurance programmes.

## Introduction

Globally, there has been a substantial decline in maternal and child mortality rates since the 1990s. In sub–Saharan Africa, for instance, maternal mortality ratio fell by approximately 45%, however, unevenly across the countries [1]. This success is largely attributed to the active policy and programmatic efforts propelled by Millennium Development Goals (MDGs) along with the efforts by numerous national and international health and development organisations[1–5]. The recognition of the fact that maternal mortality is the leading cause of death among women of reproductive age in Africa and other low-middle-income countries has been a key driver for undertaking more intensive research and funding allocation in domain of reproductive health for improving the provision and utilisation of essential maternal healthcare services (MHS)[6–8]. Despite the strengthened efforts, progress remains far from being optimum and beset by a host of issues such as inequality in access to care, regional and structural barriers to promoting the coverage of MHS. Low-middle-income countries (LMICs) still account for almost all (99%) of maternal, newborn, and child deaths occurring globally[9].

Maternal healthcare services refer to a continuum of professional check-up, educational, nutritional, and other services for pregnant woman that take place during pregnancy, partum and postpartum period[3, 10]. Ensuring proper nursing of the mother over the course of pregnancy provides the opportunities for maintaining optimum health which is a crucial determinant for healthy birth outcomes[11, 12]. Although motherhood is meant to be a rewarding and positive psychosocial experience, a considerable proportion of the women in LMICs end up seeing pregnancy and childbirth as an afflicting encounter owing to issues arising mainly during peri- and postnatal period e.g. hemorrhage, infection, hypertension, obstructed labour, preeclampsia, premature labor and birth, unsafe abortion[13–17]. Although these complications are mostly avoidable through routine uptake of MHS, a large number of women in LMICs fail to avail these services owing to various socioeconomic, cultural, and healthcare system-related barriers.

Previous studies have identified the underlying barriers to utilisation of MHS at various individual and community levels[18–20]. From a behavioural perspective, women’s healthcare seeking bahaviour can be influenced to a large extent by financial constraints and geographic distance. Various supply and demand side strategies have been proposed and implemented to promote MHS utilisation[9, 21] including home visiting, community education programmes and financial approaches e.g. maternal health insurance schemes in several Asian and African countries [22–24] including the National Health Insurance Scheme of Ghana[25, 26]. However, the impact of insurance programmes on promoting MHS has not been deeply studied to understand to what extent the increasing utilisation resulted from insurance policies, which constraints reaching conclusive findings.

A growing volume of studies suggests a positive role of health insurance on health care seeking bahaviour and health outcomes. In contrast, there is only a handful of studies assessing the influence of health insurance on MHS uptake. Countries in sub-Saharan Africa are generally characterised by low adoption of medical insurance. However, the prevalence of insurers is rising especially in the countries that have managed a modest economic growth in recent years e.g. Ghana, Nigeria[22, 27]. Ghana introduced its first National Health Insurance Scheme (NHIS) in 2005 as a demand-side strategy to promote service uptake, minimise the impact of exorbitant fees especially for the marginalized population who usually bear the highest burden of diseases and deepening poverty owing to catastrophic health expenditures [22, 28]. Several studies have attempted to identify whether the insurers are more likely to use antenatal and skilled delivery services in Ghana. However, none of the studies have focused on the types of services covered by the insurance policies on the broad range of MHS including antenatal, delivery services, and postnatal care for both mother and child. Therefore, in the present study, we aimed to investigate the differential influence of the types of services insured on MHS as well as postnatal care for the newborns. We hypothesise that having insurance coverage for a particular service will be associated with increased utilisation of that service.

## Methods

### Survey and sampling technique

Data for this study were collected from the Ghana Demographic and Health Survey (GDHS) conducted in 2014[25]. Specifically, the women and child files were used for the study. GDHS is carried out by the Ghana Statistical Service and Macro International under the auspices of DHS programs. The survey captures data on various aspects of maternal health conditions within the country and as such was deemed suitable for this study. The dataset was requested online from Measure DHS website on the 16th October, 2015. In all, 9,396 women (aged 15–49) from 11,835 households nationwide were interviewed. However, 4,294 women had birth history within the last 5 years preceding the survey and as such they constituted the sample size for this study. The 2014 GDHS was conducted with an updated frame from the 2010 Population and Housing Census (PHC) prepared by the Ghana Statistical Service (GSS). The frame exempted institutional and nomadic groups including hotel occupants and prisoners. The survey constituted a two-stage sample design for the purpose of allowing estimates of core indicators at the national level. The initial phase constituted selection of sample points (clusters) involving enumeration areas (EAs) outlined for the 2010 PHC in which 427 clusters were designated in all constituting 216 from urban and 211 from rural areas. The next stage utilised systematic sampling of households in which household inventory operation was carried out in all the identified EAs between January and March 2014.

### Variables

Three types of services within the scope of pregnancy (Timing and Adequacy of Prenatal care), delivery (Health facility delivery) and post-delivery (Postnatal care for mother and child) for the latest childbirth (occurring within preceding five years of the survey) were the dependent variables. All the variables were measured based on the answers by the individual respondents and recoded as per the WHO guidelines. For instance, ANC was categorised as timely (1) if the first visit took place within the first trimester and late (0) if otherwise; adequate (1) if made at least 4 ANC visits and inadequate (0) if otherwise. Place of delivery as Home (respondents/others home) and Health facility (hospital, clinic, health centre.). We additionally calculated the prevalence of making at least one ANC contact as a considerable percentage failed to make any ANC visit. The main explanatory variables were self-reported insurance coverage for ANC, childbirth and PNC services, which were coded as: Yes (1) and No (0).

To adjust the analysis for potential confounding variables, the following were included in the study based on the availability in the dataset and theoretical relationship with the dependent and explanatory variables: Age groups (15–24, 25–29, 30–34, 35–49); Residency (Urban, Rural); Educational status (No education, Primary, Secondary/higher); Wealth status (Poor, Non-poor); Occupation (Unemployed, Professional/technical/managerial, Sales/skilled, manual); Religion (Christian, Islam/other); Ethnicity (Akan, Ewe, Mole-Dagbani, Other); Parity (1–3, >3); Household head (Male, Female); Has health Insurance (Yes, No) [4, 18, 23, 25, 26, 28, 29]. The variables were listed and described in Table 1.

**Table 1:** Sample description. Ghana Demographic and Health Survey 2014.

Variables	Description	N = 4,293	%
**Age groups**			
15–24	Age of the respondent in the interview year	923	21.5
25–29		1058	24.6
30–34		967	22.5
35–49		1345	31.3
**Residency**	Whether the respondent is a rural or urban resident		
Urban		1778	41.4
Rural		2515	58.6
**Educational status**	Highest level of formal education attained by the respondent		
No education		1419	33.1
Primary		869	20.2
Secondary/higher		1836	42.8
**Wealth status**	Index of relative wealth status of households based on the possession of durable goods (e.g. refrigerator and TV) and building material (e.g. concrete and wooden), rather than personal income		
Poor		2241	52.2
Non-poor		2052	47.8
**Occupation**	Type of employment of the respondent		
Unemployed		767	17.9
Professional/technical/managerial		1542	35.9
Sales/skilled manual		1984	46.2
**Religion**	Type of religious affiliation of the household		
Christian		3084	71.8
Islam/other		1209	28.2
**Ethnicity**	Type of ethnic affiliation of the household		
Akan		1643	38.3
Ewe		476	11.1
Mole-Dagbani		1151	26.8
Other		1023	23.8
**Parity**	Total number of children ever born		
1–3		2505	58.4
>3		1788	41.6
**Household head**	Sex of the household head		
Male		3224	75.1
Female		1069	24.9
**Has health insurance**	Self-reported ownership of health insurance scheme		
No		1326	30.9
Yes		2967	69.1
**Early ANC**	Made ANC visit within first trimester		
No		1568	36.5
Yes		2725	63.5
**At least 1 ANC**	Made at least one ANC visit during the most recent pregnancy		
No		131	3.1
Yes		4162	96.9
**At least 4 ANC**	Made at least four ANC visits during the most recent pregnancy		
No		577	13.4
Yes		3716	86.6
**Place of delivery**	Place of delivery being respondents/relatives home of a health facility e.g. hospital, clinic, health centre.		
Home		1186	27.6
Health facility		3107	72.4
**PNC for mother**	Respondent received professional postnatal care for herself		
No		688	16.0
Yes		3605	84.0
**PNC for child**	Respondent received professional postnatal care for her child		
No		1183	27.6
Yes		3110	72.4

Household wealth status is calculated based on scores assigned to a range of household assets, for example, number of household members, floor, wall and roof material; type of cooking fuel; access to potable water and sanitation, ownership of radio, TV, refrigerator, motorcycle and others. These scores are used to perform principal components analysis that ranks the households according to their respective total scores. The final is further categorised into quintiles that classify the households into five groups from poorest to richest (Quintile 1/poorest, Quintile 2/poorer, Quintile 3/middle, Quintile 4/richer, Quintile 5/richest). Measurement of wealth index is explained in detail elsewhere[4, 29]. For health insurance, the following items were collected by the survey: Insurance covers ANC (Yes/No); Insurance covers Childbirth (Yes/No); Insurance covers mother’s PNC (Yes/No); Insurance covers child’s PNC (Yes/No); Insurance has cash benefit No (Yes/No). Analysis of the contextual items are more likely to provide a better picture of the association with the corresponding outcome variables e.g. receiving ANC. Apart from the direct coverage of the specific services, the insurance also includes a cash benefit scheme that is expected to improve the financial accessibility to the services.

### Data analysis

Data were analysed with SPSS 24. The dataset was prepared to select the variables of interest and drop the observation with missing values and outliers. Following that, the dataset was converted to adjusted for cluster design by accounting for sampling strata, primary sampling unit, and sampling weight. As the initial analysis, the basic socio-demographic characteristics of participants were presented in terms of frequencies and percentages. Prevalence of insurance ownership across the sociodemographic factors was calculated by Chi-square bivariate tests and was shown as percentages with 95%CIs. In the final step, binary logistic regression model was used to calculate the odds ratios of the associations between the measures of MHS with insurance coverage status while adjusting for the demographic and socioeconomic parameters empirically and theoretically pertinent to the outcome and exposure variables. Results of regression analysis were presented as odds ratios along with their 95%CIs as indicator of significance as well as precision of the OR values. For all associations, P-value of <0.05 was considered statistically significant.

### Ethical approval

The protocol of DHS surveys is approved by the Ethics Committee of ORC Macro Inc. The study was based on analysis of anonymised secondary data available in the public domain of DHS, therefore no additional approval was necessary. However, approval for the reuse of the data was obtained by authors from DHS.

## Results

### Descriptive analysis

Sample population comprised of 4,293 women and the mean age was 30.66 years (SD = 7.128). Basic sociodemographic characteristics of the participants were summarised in Table 1. A greater proportion of the participants were aged above 34 years, rural residents, had secondary/higher educational level, employed, followers of Christianity, of Akan ethnicity, had 1–3 children, and lived in male-headed households. Little less than two-third of the women had early ANC contact. Prevalence of at least one and at least four ANC visits were 96.9% and 86.6%, and that of facility delivery was 72.4%. respectively 84% of the women and 72% of the children had received postnatal care.

About two-third of the women reported having health insurance. Significance of the association between having health insurance across the sociodemographic variables were assessed by chi-square bivariate tests and was presented in Table 2. It shows that likelihood of having health insurance was higher among those aged above 34 years, urban residents, had secondary/higher educational level, from non-poor households, sales/skilled professional, followers of Christianity, belongs of Akan ethnicity, has 1–3 children, and from male headed households. Health insurance ownership also showed positive association with MHS and PNC for child such that women with health insurance had higher percentages of utilizing the services for themselves and children compared with those who did not have health insurance.

**Table 2:** Bivariate association between health insurance ownership with the sociodemographic variables in Ghana.

	Has health insurance		p
Variables	Yes 66.8 (64.5–68.9)	No 33.2 (31.1–35.5)	
**Age groups**			<0.001
15–24	19.9 (18.0–21.9)	24.6 (21.9–27.4)	
25–29	25.9 (24.0–27.9)	20.8 (18.5–23.3)	
30–34	24.0 (22.1–26.0)	22.2 (19.3–25.5)	
35–49	30.3 (28.1–32.6)	32.4 (29.4–35.5)	
**Residency**			<0.001
Urban	52.5 (48.5–56.6)	56.3 (51.6–60.9)	
Rural	47.5 (43.4–51.5)	43.7 (39.1–48.4)	
**Educational status**			<0.001
No education	26.5 (22.8–30.6)	25.8 (22.5–29.4)	
Primary	24.5 (21.8–27.5)	17.2 (15.4–19.1)	
Secondary/higher	48.9 (44.7–53.2)	57.0 (53.5–60.5)	
**Wealth status**			0.007
Poor	39.6 (35.8–43.5)	44.7 (40.0–49.5)	
Non-poor	60.4 (56.5–64.2)	55.3 (50.5–60.0)	
**Occupation**			<0.001
Unemployed	18.5 (16.6–20.6)	15.9 (13.8–18.1)	
Professional/technical/ managerial	30.5 (27.8–33.3)	31.9 (27.9–36.3)	
Sales/skilled manual	51.0 (48.1–53.9)	52.2 (47.9–56.5)	
**Religion**			0.003
Christian	75.4 (71.4–79.0)	78.7 (75.1–81.9)	
Islam/other	24.6 (21.0–28.6)	21.3 (18.1–24.9)	
**Ethnicity**			<0.001
Akan	44.1 (40.2–48.2)	53.9 (49.1–58.6)	
Ewe	14.3 (12.1–16.8)	11.1 (8.9–13.9)	
Mole-Dagbani	19.2 (15.4–23.5)	13.6 (11.0–16.6)	<0.001
Other	22.4 (19.0–26.2)	21.4 (17.4–26.1)	
**Parity**			0.001
1–3	62.9 (60.8–65.1)	56.9 (53.6–60.1)	
>3	37.1 (34.9–39.2)	43.1 (39.9–46.4)	
**Household head**			<0.001
Male	75.3 (72.6–77.8)	67.4 (64.1–70.6)	
Female	24.7 (22.2–27.4)	32.6 (29.4–35.9)	
**Early ANC**			<0.001
No	33.7 (30.8–36.8)	40.4 (36.9–44.1)	
Yes	66.3 (63.2–69.2)	59.6 (55.9–63.1)	
**At least 1 ANC**			<0.001
No	1.3 (0.8–2.1)	5.1 (3.6–7.2)	
Yes	98.7 (97.9–99.2)	94.9 (92.8–96.4)	
**At least 4 ANC**			<0.001
No	8.9 (7.6–10.5)	18.9 (15.6–22.7)	
Yes	91.1 (89.5–92.4)	81.1 (77.3–84.4)	
**Place of delivery**			<0.001
Home	21.0 (18.7–23.5)	31.2 (27.2–35.5)	
Health facility	79.0 (76.5–81.3)	68.8 (64.5–72.8)	
**PNC for mother**			<0.001
No	11.8 (9.7–14.2)	17.4 (14.3–21.0)	
Yes	88.2 (85.8–90.3)	82.6 (79.0–85.7)	
**PNC for child**			<0.001
No	26.3 (23.1–29.7)	30.4 (25.9–35.3)	
Yes	73.7 (70.3–76.9)	69.6 (64.7–74.1)	

**Fig 1:**
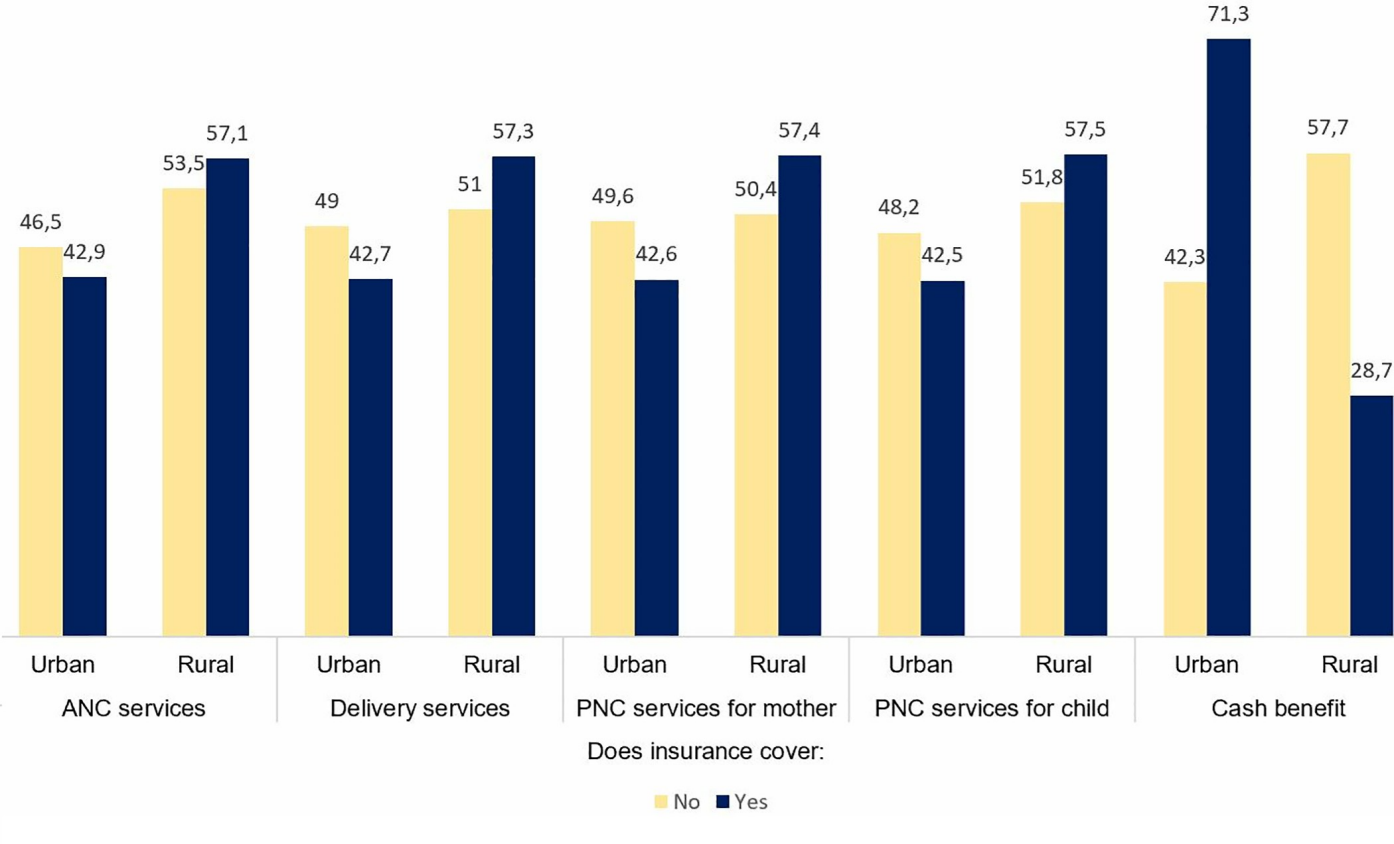
Types of maternity services covered by health insurance in Ghana, 2014.

Fig 1 shows the percentage of types of services under the coverage of health insurance in urban and rural areas. In general, insurance schemes in urban areas had lower percentage of covering ANC, childbirth and postnatal services than in rural, but the differences were not statistically significant. However, urban women enjoy a significantly higher percentage of cash benefits within their insurance schemes compared with rural women.

Table 3 summarises the results of cross-tabulation between the types of services covered by health insurance and the respective percentage of utilizing that service. In general, having insurance for a particular type of services showed a higher percentage of uptake of that service. However, some exceptions were observed for certain combination of insurance coverage and service uptake. Having covered for ANC services showed higher percentages for making at least one and four ANC visits, but not on making early ANC contact, delivering at a health facility, and receiving PNC for mother.

**Table 3:** Percentage of service uptake by insurance coverage status.

	Early ANC		1 ANC		4 ANC		Facility delivery		PNC for mother		PNC for child	
	No	Yes	No	Yes	No	Yes	No	Yes	No	Yes	No	Yes
**Insurance covers ANC**												
No	30.8	69.2	3.8	96.2	15.1	84.9	22.0	78.0	12.6	87.4	39.6	60.4
Yes	34.7	65.3	1.6	98.4	10.0	90.0	23.9	76.1	14.1	85.9	24.9	75.1
**Insurance covers Childbirth**												
No	32.8	67.2	3.0	97.0	15.2	84.8	24.2	75.8	13.1	86.9	37.4	62.6
Yes	34.6	65.4	1.6	98.4	10.0	90.0	23.7	76.3	14.1	85.9	24.9	75.1
**Insurance covers mother’s PNC**												
No	32.2	67.8	2.5	97.5	12.3	87.7	22.0	78.0	13.6	86.4	44.9	55.1
Yes	34.7	65.3	1.6	98.4	10.1	89.9	23.9	76.1	14.1	85.9	24.0	76.0
**Insurance covers child’s PNC**												
No	31.5	68.5	2.9	97.1	11.9	88.1	23.2	76.8	14.1	85.9	42.1	57.9
Yes	34.9	65.1	1.5	98.5	10.1	89.9	23.8	76.2	14.0	86.0	23.8	76.2
**Insurance has cash benefit**												
No	34.9	65.1	1.7	98.3	10.4	89.6	24.3	75.7	14.3	85.7	25.7	74.3
Yes	23.0	77.0	1.1	98.9	9.2	90.8	4.6	95.4	4.6	95.4	25.3	74.7

Table 4 summarises the results of the multivariate association between the types of MHS with insurance coverage status. It indicated that women who had their ANC services covered had significantly higher odds of attending at least one and four ANC visits, as well as receiving PNC for the child. Notably, insurance coverage for childbirth services was associated with higher odds of delivering at a health facility, however, the association was not statistically significant, indicating the presence non-financial factors on women’s preference for venue of delivery. Having insurance for PNC services for mothers showed significantly higher odds (OR = 1.316, p<0.05) of receiving PNC for children, but not for mothers (OR = 0.863, p<0.05). Having cash benefit showed a significantly positive association with making early ANC visit (OR = 2.046, p<0.05), facility delivery (OR = 1.449, p<0.05), and PNC for mother (OR = 1.290, p<0.05).

**Table 4:** Odds ratio of MHS uptake by insurance coverage status in Ghana.

	Early ANC	1 ANC	4 ANC	Facility delivery	PNC for mother	PNC for child
**Insurance covers ANC (No)**						
Yes	0.844	1.846*	1.178*	0.767	1.080	1.658*
**Insurance covers Childbirth (No)**						
Yes	0.894	1.172	1.340*	1.351	0.907	2.036*
**Insurance covers mother’s PNC (No)**						
Yes	1.571	0.986	1.276	0.864	1.106	1.515*
**Insurance covers mother’s PNC (No)**						
Yes	1.215	4.337	2.782	1.116	0.863	1.316*
**Insurance has cash benefit (No)**						
Yes	2.046*	1.177	0.740	1.449*	1.290*	0.702

N.B. (No) = reference category.

* denotes statistically significant results.

Model quality was assessed by using pseudo-R^2^ statistics, and the values ranged from .239 to .403 which indicated a satisfactory model fit.

## Discussion

This study reports the prevalence of insurance ownership among women across various demographic and socioeconomic backgrounds, as well as the association between insurance ownership and utilisation of antenatal, professional delivery, and postnatal care for mothers and children. Overall, about two-third of the women reported having health insurance in 2014, compared with two-fifth in 2008[22]. Analysing by the background characteristics revealed that women from urban areas, with higher education and wealth status, were the ones more likely to have health insurance. In general, insurance programmes in urban areas had a lower percentage of covering for ANC, childbirth and postnatal services than in rural, with urban women having a significantly higher percentage of cash benefits within their insurance schemes compared with rural women. Addressing the socioeconomic gap in insurance ownership is likely to remain a challenge in the sub-Saharan African countries where a substantial proportion of the population live below poverty line and are least capable of accessing healthcare services, despite sharing higher health risks and disease burden.

A recent study on health insurance policies in five sub-Saharan countries (Ghana, Tanzania, Kenya, Rwanda, and Ethiopia) has concluded that the existing programmes are leaving out the most marginalized, including Ghana, where many of the targeted services are not within the reach of the poor[30]. So far there are no large-scale researches focusing on the extent to which the most marginalised population are benefiting from the insurance programmes, especially in the context of reproductive and MHS. Many countries in sub-Sahara Africa have been able to increase the uptake of MHS and reduce maternal and child mortality rates. However, review of the literature suggests that the progress has not been even across and within countries and is occurring mostly among the relatively wealthier sections of the population. Therefore, it is essential that health policymakers pay special attention to the marginalised population and investigate the efficacy of the insurance and cash benefit programmes to promote MHS utilisation. In Ghana there has been an increasing percentage of population under various health and social insurance programmes and subsequent improvement in MHS uptake, as also supported by the present findings.

In general, having insurance for a particular type of services showed a higher percentage of uptake of that service. The positive impact of health insurance ownership on MHS utilisation was reported in Kenya, Rwanda and Tanzania[22, 23, 31]. However, some exceptions were observed in the present study for a certain combination of insurance coverage and service uptake. For instance, having covered for ANC services showed higher percentages for making at least one and four ANC visits, but not on making early ANC contact, delivering at a health facility, and receiving PNC for mother. The impact of health insurance on promoting the utilization of MHS is likely to vary depending on the characteristics, such as the amount of premiums, benefits plans, location and quality of healthcare services[32, 33]. In the present study, we had no information on whether the premiums were exempted and state of healthcare services, so it is possible that the inclusion of these variables could alter the strength of the associations. Regardless of that, we found that insurance coverage for specific types of MHS was generally associated with higher likelihood of utilising respective services. Some exceptions included early initiation of ANC, and choice of place of delivery. Having insurance for ANC care showed positive association with the number of minimum one adequate ANC visits, but it had no influence on the timely starting of visits. Insurance coverage for childbirth services had positive association with having adequate number of ANC visits, but not on venue of delivery. Of note, preference for venue of delivery was not influenced by having insurance for childbirth either. This finding partly reflects the influence of non-pecuniary factors that have shown to play strong roles in health perception and healthcare-seeking behaviour of individuals. However, offering cash benefits was associated with increased odds of choosing facility delivery, implying the direct cash offers can act as better motivating factors compared with insurance coverage for a particular service. More studies should be carried out to explore the relative benefits of various insurance schemes and ensure the efficacy of the existing interventions strategies.

## Conclusion

Despite the increasing prevalence of having health insurance ownership and utilising essential maternal healthcare services, socioeconomic inequality in insurance ownership is still common. Our findings also indicate that comprehensive and equitable expansion of health insurance enrollment can lead to higher rates of maternal care utilization in Ghana. However, the results need to be interpreted with caution as the survey was cross-sectional and lacked information on behavioural and cultural factors that might have influenced the estimation of the positive effects of health insurance. Future researches should focus on exploring the causes of lower insurance coverage among certain population and analyse the effectiveness of health insurance programmes in promoting MHS among different socioeconomic groups.
